# Intermuscular Coherence in the Presence of Electrical Stimulation

**DOI:** 10.3389/fnsys.2021.647430

**Published:** 2021-05-04

**Authors:** Jonathan A. Norton

**Affiliations:** Division of Neurosurgery, Department of Surgery, University of Saskatchewan, Saskatoon, SK, Canada

**Keywords:** coherence, electrical stimulation, sensory feedback, muscle, oscillation

## Abstract

The nervous system uses oscillations to convey information efficiently. Inter-muscular coherence in the 15–35 Hz range is thought to represent common cortical drive to muscles, but is also in the frequency band in which electrical stimulation is applied to restore movement following neurological disease or injury. We wished to determine if, when stimulation is applied at the peak frequency of the coherence spectra it was still possible to determine voluntary effort. Using healthy human subjects we stimulated muscles in the arms and legs, separate experiments, while recording EMG activity from pairs of muscles including the stimulated muscles. Offline coherence analysis was performed. When stimulation is greater than motor threshold, and applied at the peak of the coherence spectra a new peak appears in the spectra, presumably representing a new frequency of oscillation within the nervous system. This does not appear at lower stimulation levels, or with lower frequencies. The nervous system is capable of switching oscillatory frequencies to account for noise in the environment.

## Introduction

Neural information is conveyed through several features of the firing pattern of the nervous system, or portions thereof (Soteropoulos and Baker, 2009). Frequency encoding of information is used in many areas of the nervous system and is an efficient mechanism to transmit information. Coherence is the equivalent of cross-correlation in the frequency domain (Challis and Kitney, 1991; Halliday and Rosenberg, 1999). Farmer et al. (1993) showed that coherence between motor units was common in frequency band between 16 and 32 Hz. Numerous studies have shown that there is cortico-muscular coherence in the beta-band (β-band, 15–35 Hz) using electrodes in the cortex and muscle (Kilner et al., 1999; Mima and Hallet, 1999). Coherence between or within muscle at these frequencies represents common cortical drive to the muscle(s). Such coherence is part of a closed-loop feedback circuit that relies in part on sensory nerve fibers. Subject IW, with large fiber poly-neuropathy, has altered coupling between muscles, but still shows peaks in the 16–32 Hz band between motor units (Farmer et al., 1993; Kilner et al., 2004). Cooling the limb to slow conduction velocity affects the coherence (Riddle and Baker, 2005). The IA afferent system can be accessed through muscle vibration, and 20 Hz muscle vibration has previously been shown (Farmer et al., 1997) to drive coherence between motor units in a given muscle. In this study we wished to determine if external, open-loop electrical stimulation affected β-band intermuscular coherence.

Open-loop stimulation may be used for research studies, but more commonly as a neuroprosthetic aid (Peckham and Knutson, 2005). Even in closed-loop systems, the frequency of the stimulation is often invariant, that is open-loop. We have previously speculated on the use of intermuscular coherence as a control source for a Functional Electrical Stimulation (FES) device, for cycling (Lou et al., 2010), but extendable to other systems. We thought that by using β-band coherence as a measure of voluntary effort we may be able to promote neural recovery when using the device. The ideal stimulation frequency for a FES device is a trade-off between reducing muscle fatigue and ensuring smooth muscle contractions. A typical device may stimulate the nerve/muscle at around 20 Hz therefore (Rushton, 1997). This is close to the peak frequency in the β-band in the coherence plots in many subjects. The advantage of working in the frequency domain in the presence of large stimulation artifacts, especially with relatively short inter-stimulus intervals (50 ms for 20 Hz stimulation) is that the artifact is constrained to a single frequency (with harmonics) whereas in the time domain the effects of the stimulus can last for several times the interstimulus interval (Thorsen, 1999; Norton et al., 2004). In this study we wished to determine if stimulating at the peak frequency of coherence for that individual, in a given task, altered the intermuscular coherence, such that the coherence might still be a usable control signal.

Cortical control of upper limb and lower limb tasks differs, with an increased focus on sub-cortical control in the lower limb, walking for example being based upon spinal pattern generators (Kiehn et al., 2010). In contrast many upper limb tasks require fine motor control and are tightly controlled by the motor cortex (Lemon, 2008). Cortical representation of the upper limb is larger than that of the lower limb. We wished to determine if our findings were generalizable across limbs and tasks.

The aim of the study was to electrically stimulate and record muscle activity at a fixed frequency corresponding to the peak in the intermuscular coherence for that subject and determine if the coherence plot changed.

## Methods

All experiments were approved by the Human Research Ethics Board of the University of Saskatchewan. All subjects in this study were neurologically normal. Some subjects took part in both phases of the study whilst most just did either upper or lower limb studies. Each trial had 15 participants, nine female for both studies. The age range for the upper limb was 21–58 (mean 34) years of age and for the lower limb was 19–55 (mean 37). All subjects were tested on their dominant side (13 right for upper limb and 15 right for lower limb, we asked subjects to identify dominant leg for lower limb studies).

### Tasks

The tasks were divided into a static hold and a functional task for both the upper and lower limb. For the static hold subjects were asked to maintain an isometric contraction of about 15% MVC using smoothed, rectified EMG as feedback (details on EMG recording below). For the functional task in the upper limb subjects held a cup of water and took it from the table to their lips repeatedly. In the lower limb subjects walked on a treadmill at a self-selected walking speed. All recordings were of 2 min duration.

### Recordings

Surface EMG electrodes (Ag/AgCl) were used in all recordings and were placed on the skin overlying the muscle following skin cleaning and preparation. Skin resistances were <5 kΩ. EMG signals were amplified and filtered (3–3 kHz) (Neurolog, Digitimer, Welwyn Garden City, United Kingdom) before being sampled at 1 kHz (CED Micro1401, Cambridge Electronic Design, Cambridge, United Kingdom) and stored on a PC running Spike2 (also CED). Feedback EMG was low pass filtered at 20 Hz and displayed on an oscilloscope to the subject. Stimulation was provided by a DS7A (Digitimer). Stimulating electrodes were placed more between the pair of recording electrodes to generate a worst-case scenario for artifact. For the upper limb recordings were taken from flexor carpi radialis and ulnaris and for the lower limb from tibialis anterior and vastus lateralis. Muscle pairs were chosen to have periods of overlapping contraction during the tasks (holding a cup and walking). Stimulation was applied to the flexor carpi radialis and tibialis anterior, respectively. The axes of the recording and stimulating dipoles were aligned and placed on the midline of the muscle belly.

### Study Protocol

**FIGURE 1:**
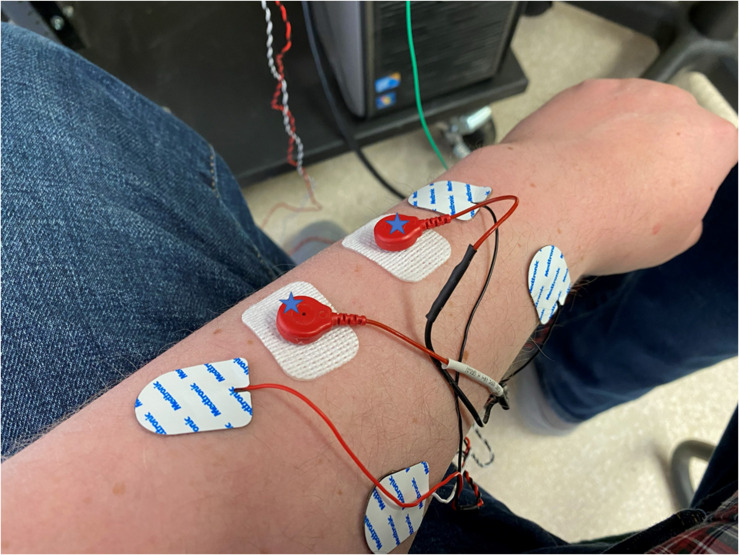
The placement of electrodes on the forearm of a subject with the stimulation electrodes being the white rectangular electrodes, marked with a star, and the recording electrodes being rounder with a built-in cable. Stimulating and recording electrodes are placed in close proximity to each other and aligned parallel to the muscle fibers.

All of the tasks followed the same arrangement. Subjects completed two sets of the task in the absence of stimulation. Following this the intermuscular coherence was calculated. Stimulation was then applied at twice sensory threshold at 10% of the peak frequency in the coherence plot, again this was done two times. The intermuscular coherence was calculated to confirm the peak frequency had not shifted. Stimulation was then applied at that frequency at 90% sensory threshold, 110% sensory threshold and 200% sensory threshold or 110% motor threshold whichever was high. The order of these stimulations was randomized, and each test was performed two times for both the static and dynamic tasks. The coherence was not re-calculated during these trials. To account for differences in relative placement of the electrodes to the nerve, skin resistance, etc., we report stimulation levels in terms of sensory and motor thresholds and not measured current. Figure 1 illustrates the placement of the electrodes on the upper limb with the stimulation electrodes placed between the recording electrodes.

In the upper limb only, we also examined the effect of 20 Hz stimulation in all subjects (unless 20 Hz was the dominant peak identified earlier) in order to determine whether any changes were only seen when the dominant peak frequency is stimulated or whether it was a feature of the faster stimulation. This experiment was performed after the initial findings, and so only supra motor threshold stimulation and no-stimulation conditions were tested. This is similar to the experiment of Farmer et al. (1997) in which 20 Hz vibration was applied to the muscle.

### Walking

During walking the tibialis anterior muscle is only active for approximately 40% of the gait cycle (Whittle, 1996). We asked subjects to walk for long enough that, when concatenated, we would have 2 min of active tibialis anterior muscle activity. Concatenation and windowing was performed as previously (Norton and Gorassini, 2006).

### Analysis

As described above, surface EMG was collected using Spike2 and then transferred to Matlab where it was rectified and the coherence and phase calculated, using scripts based on those developed by the Neurospec group^1^ (Halliday et al., 1995). The resolution of the spectral plots was 0.96 Hz and 95% confidence limits were calculated as described previously (Amjad et al., 1997; Halliday and Rosenberg, 1999). The peak frequency as determined by visual inspection as the frequency corresponding to the highest peak in the β-band in the coherence plot. Phase plots were used to confirm that the coherence was not random. Comparison of the peak frequencies with and without stimulation was performed. Our null hypothesis was that there would be no change in the peak frequency of coherence (δF = 0).

## Results

All subjects tolerated all aspects of the stimulation and completed the portion of the study they enrolled in. At baseline all subjects showed a peak in the coherence plots within the β-band (15–35 Hz) that was in the frequency band 19–28 Hz.

**FIGURE 2:**
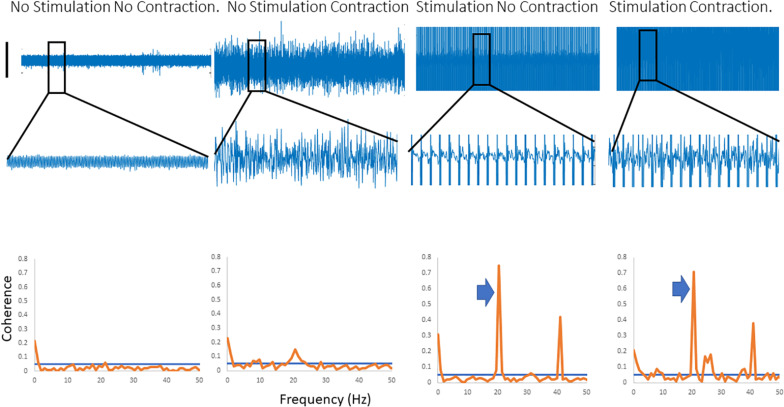
Four columns of data from the arm are shown in this figure. Row 1 has raw EMG from the stimulated muscle, while row 2 highlights an expanded portion of that EMG, and row 3 shows coherence between the stimulated and the unstimulated, synergistic muscle. In the first column there is no activity, and hence no coherence. The second column has a background contraction, but no stimulation while the third column has stimulation but no contraction. The fourth column shows both stimulation and contraction simultaneously. In the coherence plots the arrows indicate the peak in the coherence as a result of the stimulation.

**FIGURE 3:**
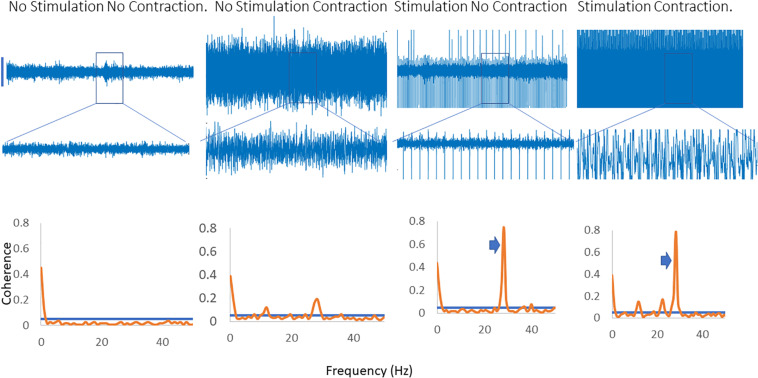
Four columns of data from the leg are shown in this figure. Row 1 has raw EMG from the stimulated muscle, while row 2 highlights an expanded portion of that EMG, and row 3 shows coherence between the stimulated and the unstimulated, synergistic muscle. In the first column there is no activity, and hence no coherence. The second column has a background contraction, but no stimulation while the third column has stimulation but no contraction. The fourth column shows both stimulation and contraction simultaneously. In the coherence plots the arrows indicate the peak in the coherence as a result of the stimulation.

Figure 2 shows traces obtained from the upper limb with raw EMG, expanded raw EMG from one muscle and coherence between muscles in each of four conditions; rest, background contraction, stimulation alone and stimulation and contraction. Similar data is shown for the leg in the Figure 3. In both figures only one peak is visible in the plots in the β-band except in the stimulation and contraction condition (right hand column).

The difference in frequency (δF) between the two peaks in F was the primary outcome measure. No change in frequency was seen when stimulation was applied below motor threshold. Only supra-motor threshold data (two times sensory threshold) is presented here.

Some subjects showed an increase in peak frequency, whilst others showed a decrease. To prevent the changes canceling each other out the magnitude of the change was used in statistical analysis rather than the raw value. These inter subject differences make the application of pooled coherence estimates difficult to justify.

There was no difference in peak frequency of coherence in the no-stimulation and low stimulation intensity condition (0.19 ± 0.41 Hz for the upper limb and 0.32 ± 0.49 Hz for the lower limb. *p* > 0.2, paired *t*-test) or between the low frequency stimulation and no stimulation condition (0.06 ± 0.25 Hz for the upper limb and 0.38 ± 0.5 Hz for the lower limb, *p* > 0.2, paired *t*-test). Between high frequency stimulation and no stimulation there was a statistically significant difference across all tasks (4.4 ± 1.1 Hz for the upper limb and 5.5 ± 1.4 Hz for the lower limb, *p* < 0.05, paired *t*-tests).

Directional analysis indicated that in the absence of stimulation neither muscle in the upper limb led the other, while in the lower limb the more proximal muscle appeared to lead the more distal by 4 ms. When stimulation was applied the stimulated muscle led the other (distal led proximal in the leg) by 30 ms.

**FIGURE 4:**
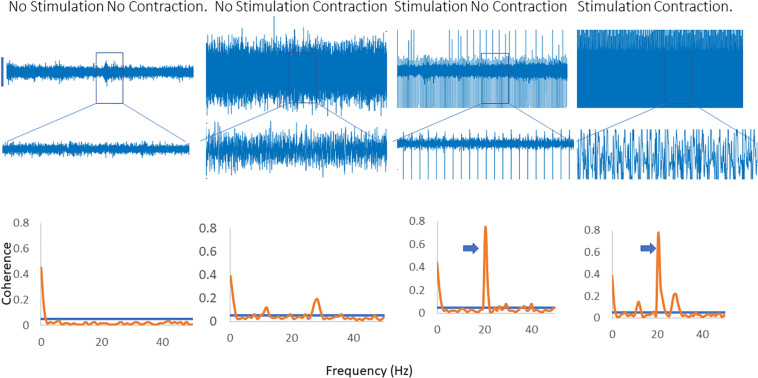
Inter-muscular coherence from the upper limb is shown with a background contraction and no stimulation, a contraction and 20 Hz stimulation and a contraction with stimulation at the peak frequency in the coherence spectra. In the 20 Hz stimulation contraction there is a peak in the coherence at 20 Hz along with the original peak in the coherence spectra. The trace shows the effects of stimulating at the peak frequency of the background coherence spectra. A new peak emerges at a lower frequency in the spectra. In the coherence plots the arrows indicate the peak in the coherence as a result of the stimulation.

Figure 4 illustrates the effects of applying a fast (20 Hz) rate of stimulation when that is not the dominant peak in the coherence spectra. A clear peak is seen at 20 Hz, with a harmonic at 40 Hz and a single, broader peak is identified which is present with and without stimulation and does not change in frequency.

## Discussion

We have shown that in the presence of fixed frequency electrical nerve stimulation the nervous system is able to produce coherence at a new peak frequency. The frequency of this peak is consistent between trials and tasks for an individual. Intermuscular coherence may be a way to control neural protheses in the future, but work is still needed to relate the peak in the frequency to the voluntary effort in the presence of electrical stimulation.

Fixed frequency electrical stimulation is novel to the nervous system. Most of the subjects had not taken part in electrical stimulation experiments previously and even those who had it would have been a negligible amount of time over their lifetime. Coherence is the result of a closed-loop within the nervous system, that is, there is feedback to the generator of the oscillations (Pohja and Salenius, 2003; Riddle and Baker, 2005). Because of the feedback it is possible for the oscillations to be changed because of what is taking place in the periphery. This study suggests that the nervous system monitors the feedback from the oscillations and compares the results with those expected, as it does with other motor outputs. Theoretically the nervous system may increase the size of the oscillations to “overcome” the disturbance of the electrical stimulation, but at least in the study we found that the oscillations change frequency. This is a lower energy requirement and more efficient method of communication than increasing the amplitude of the oscillations. We find it interesting that the nervous system appears to have a solution to a problem it has never seen before. To our knowledge this is the first time such an observation has been made.

Because the effect is only seen when the stimulation is applied and there is a voluntary contraction of the muscle, and not when stimulation is applied alone or at a different, but similar, frequency we believe that the effect, a new peak in the coherence plot, is not an artifact of the stimulation. Notably significantly more energy is injected into the system with the faster stimulation rate. However, the change in frequency is only seen when stimulation is applied at the dominant frequency of the coherence spectra not at when 20 Hz stimulation is arbitrarily applied. In these cases, the amount of energy injected into the system is very similar, and both stimulation protocols (20 Hz and peak frequency) were sufficiently fast to generate a smooth muscle contraction.

For an efficient controller for neural prostheses that might maximize recovery the input to the controller should be proportional to the effort of the subject (Rushton, 2003). We have not demonstrated that in this project, but we have shown that the peak frequency of coherence in the presence of supra-motor threshold stimulation is recordable. This is a first step toward the goal of this type of controller. We have been working with peripheral nerve stimulation applied on the surface, the most common form (Taylor et al., 1999) but also the one with the largest stimulation currents. Implanted systems (muscular, peripheral nerve, or spinal column) are likely to have smaller electrical artifacts (Donaldson et al., 1997; Grill et al., 2009; Harkema et al., 2011), but may induce the same change in peak frequency.

The limitations of this study include that the trials were performed on the same day, so we do not know if subjects switch to the same frequency on a consistent basis. We asked subjects to provide a constant background contraction and so we do not know how effort affects the peak of the coherence. We were not able to stimulate at a maximal level that may be needed in a very paretic patient. Indeed, all of the subjects in this study were neurologically normal and we will need to demonstrate that the same effect is present in those with both spinal and cortical lesions for this to be a functional system. Future experiments will also involve the recording of EEG to better determine the site of the changes in frequency and include stochastic stimulation.

## Data Availability Statement

The raw data supporting the conclusions of this article will be made available by the authors, without undue reservation.

## Ethics Statement

The studies involving human participants were reviewed and approved by the University of Saskatchewan Human Research Ethics Board. The patients/participants provided their written informed consent to participate in this study.

## Author Contributions

JN conceived the study, carried out the experiments, performed the analysis, and wrote and edited the manuscript.

## Conflict of Interest

The author declares that the research was conducted in the absence of any commercial or financial relationships that could be construed as a potential conflict of interest.

## Footnotes

1 www.neurospec.org
